# A Rare Case of Left Main Coronary Artery Coronary Sinus Fistula in an 85-Year-Old Female

**DOI:** 10.7759/cureus.61320

**Published:** 2024-05-29

**Authors:** Hanad Bashir, Ahmed A Ahmed, Mohammad Akhtar, Amanda R Beering, Teresa M Ratajczak

**Affiliations:** 1 Cardiovascular Medicine, The Christ Hospital, Cincinnati, USA; 2 Internal Medicine, Rochester Unity Hospital, Rochester, USA; 3 Internal Medicine, The Christ Hospital, Cincinnati, USA

**Keywords:** angiogram, congenital heart disease, atrial fibrillation, angina, dyspnea, ct angiography, left main coronary artery, coronary sinus fistula, coronary artery disease, arteriovenous malformations

## Abstract

Coronary arteriovenous fistulas (CAVFs) are congenital or acquired communications between the coronary arteries and coronary venous system, and they can also include other cardiac structures or vasculature. We discuss a case of a large fistula between the left main coronary artery and the right atrium in a geriatric patient with a history of gastrointestinal arteriovenous malformations (AVM). The occurrence of CAVFs, an uncommon cardiac irregularity, is particularly infrequent among older adults. Typically, it is discovered by chance when investigating symptoms such as shortness of breath or chest pain, where coronary angiography is necessary to determine the most effective treatment strategy. This case highlights the possible utility of evaluating CAVFs in patients with a history of gastrointestinal AVM who similarly present with clinical symptoms of high-output heart failure. Once identified, this could simplify the treatment approach and improve communication between healthcare providers to minimize the risk of harm to the patient.

## Introduction

Coronary arteriovenous fistulas (CAVFs) are rare cardiac anomalies, representing 0.2%-0.4% of congenital heart defects and about 0.2% of coronary angiographies. They are most commonly diagnosed in adults, typically in their 50s and 60s, and affect males and females equally. There is no significant predilection for any particular race or ethnicity as CAVFs have been reported across diverse populations. Due to their often asymptomatic nature, these fistulas are frequently discovered incidentally during diagnostic procedures for other cardiovascular issues. They are categorized based on their communication type with the cardiac chamber, either arterio-luminal (direct communication) or arterio-sinusoidal (communication through the sinusoidal network). Roughly 90% of these abnormalities drain into the right-sided chamber or a major vessel [1]. This can cause fistula-related ischemia known as the “coronary steal phenomenon," whereby blood is shunted away from the coronary circulation and causes volume overload in both ventricles. Eventually, this leads to congestive heart failure, pulmonary hypertension, and ischemia due to increased myocardial oxygen demand. Additionally, the potential link between gastrointestinal AVMs and coronary AVMs is not well-documented, and there is limited understanding of the mechanisms underlying post-natal AVM formation. Despite the available data, several aspects of CAVFs remain poorly understood [2,3]. Herein, we describe a case of a large fistula between the left main coronary artery and the right atrium in a geriatric patient with a history of gastrointestinal arteriovenous malformations (AVM).

## Case presentation

An 85-year-old female presented with several cardiac risk factors, which included hypertension, age, obesity, and chronic diastolic heart failure. She also has a past medical history of colon cancer status post partial colon resection and lower gastrointestinal bleeding due to multiple AVM identified in the right colon on colonoscopy requiring argon plasma coagulation (APC). The patient required two units of red blood cell transfusion and was also on Aspirin 81 mg oral at the time of APC. She is also suspected to have chronic obstructive pulmonary disease but has no record of completing a pulmonary function test and was referred for outpatient evaluation of newly diagnosed atrial fibrillation with a controlled ventricular response identified on an electrocardiogram (EKG).

A few weeks before her presentation, she began experiencing acute onset chest heaviness and dyspnea upon exertion, which slightly improved with albuterol 90 mcg (two puff inhalation as needed). She reported two pillow orthopnea but slept in a recliner the majority of the time. She also mentioned gastroesophageal reflux disease (GERD)-like symptoms and dysphagia on follow-up visits. Vital signs were within normal limits and body mass index (BMI) was 30 kg/m^2^. The patient’s heart sounds were irregularly irregular with bilateral lower extremity edema noted on physical examination. Recent blood work from outside hospitalization showed hemoglobin of 11.3 grams/deciliter. The lipid panel, hemoglobin A1c, liver function test, and basic metabolic panel were all unremarkable. A Lexiscan nuclear stress test with nuclear imaging was ordered to evaluate cardiac ischemia, and anticoagulation was withheld in the setting of her history of lower gastrointestinal bleeding from AVM. She was started on diltiazem 120 mg (one tablet by mouth daily), and her previously prescribed amlodipine 5 mg (one tablet by mouth daily) was discontinued.

**Figure 1:**
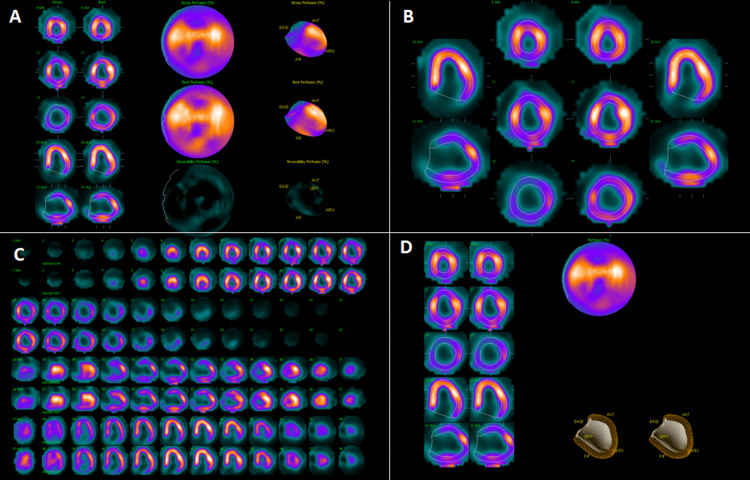
(A) Nuclear imaging showing reversible ischemia in basal lateral on horizontal long axis view and basal inferior on vertical long axis view. (B) Reversible ischemia was noted in the basal inferior and basal lateral view of the left ventricle as well as global ischemia on the short axis basal view. (C) Reversible ischemia was noted in the basal inferior and basal lateral portion of the left ventricle with global ischemia at the base. (D) Reversible ischemia was noted in the basal lateral and inferior basal portion of the left ventricle with global ischemia at the base.

The Lexiscan nuclear stress test showed a small area of infarct and insignificant peri-infarct ischemia in the region supplied by the left anterior descending artery (LAD) and diagonal branches (Figure 1). The left ventricular ejection fraction could not be estimated due to atrial fibrillation. Upon follow-up, the patient was started on furosemide 20 mg (one tablet by mouth every other day) for dyspnea upon exertion. The patient was referred to the structural heart clinic for evaluation for left atrial appendage closure through a WATCHMAN procedure as anticoagulation was contraindicated.

**Figure 2:**
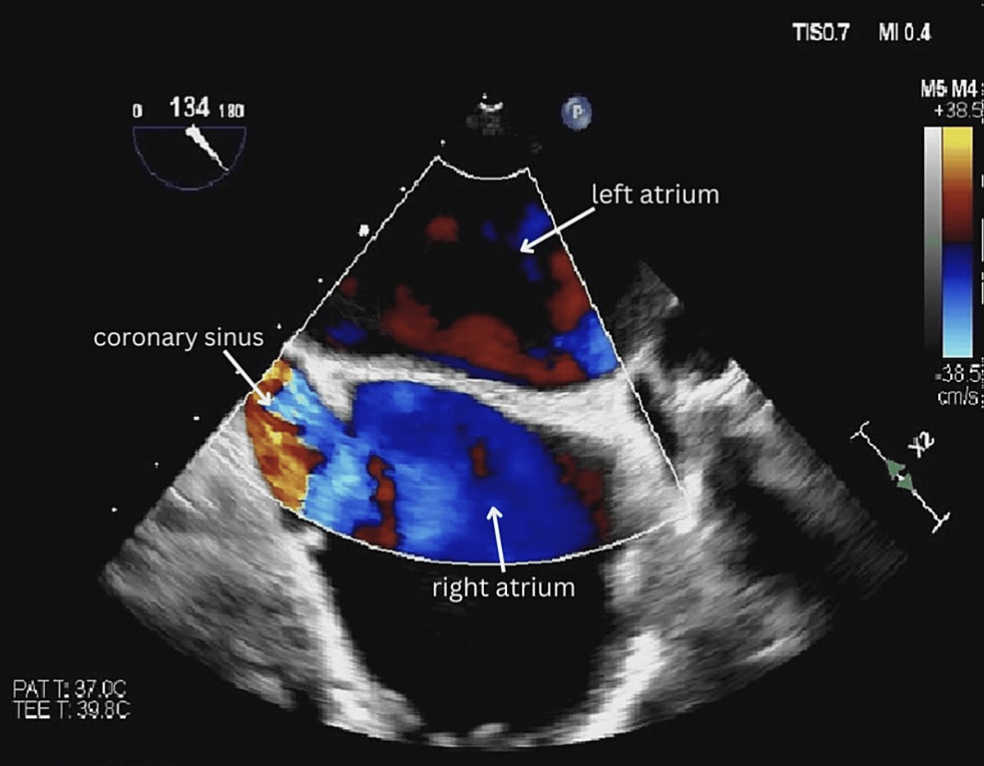
Transesophageal echocardiogram mid-esophageal bicaval view showing systolic flow acceleration from the coronary sinus into the right atrium

The patient was referred to the structural heart clinic for evaluation of left atrial appendage closure through a WATCHMAN device. This minimally invasive procedure is non-inferior to warfarin and is a safe alternative for the prevention of stroke and systemic embolization related to non-valvular atrial fibrillation in settings where anticoagulation is contraindicated [4]. The transesophageal echocardiograms (TEE) revealed a dilated left main coronary artery with a large anomalous vascular structure, which was suspected to be branching from the left main coronary artery but ultimately could not be identified. Systolic flow with an aortic waveform was observed within this structure along with low-velocity diastolic flow (Figure 2).

**Figure 3:**
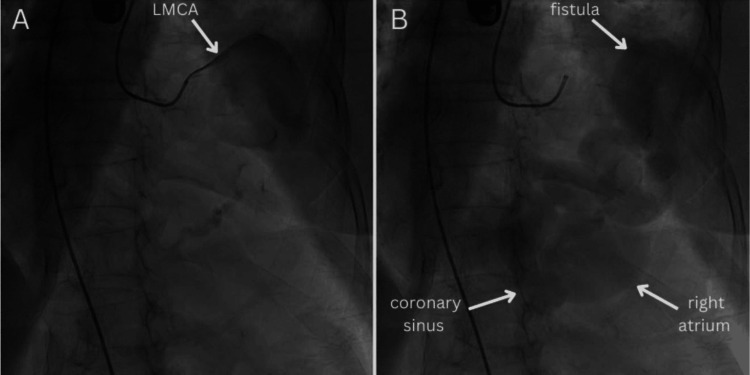
(A,B) The coronary angiography of the left coronary system shows a large dilated fistula that connects the left main coronary artery (LMCA) to the right atrium, which is consistent with the coronary sinus.

As a result, it was decided to perform coronary angiography, which showed a very dilated fistula arising from the left main coronary artery and flowing into the right atrium consistent with the coronary sinus. These findings matched the pre-procedural TEE (Figure 3).

**Figure 4:**
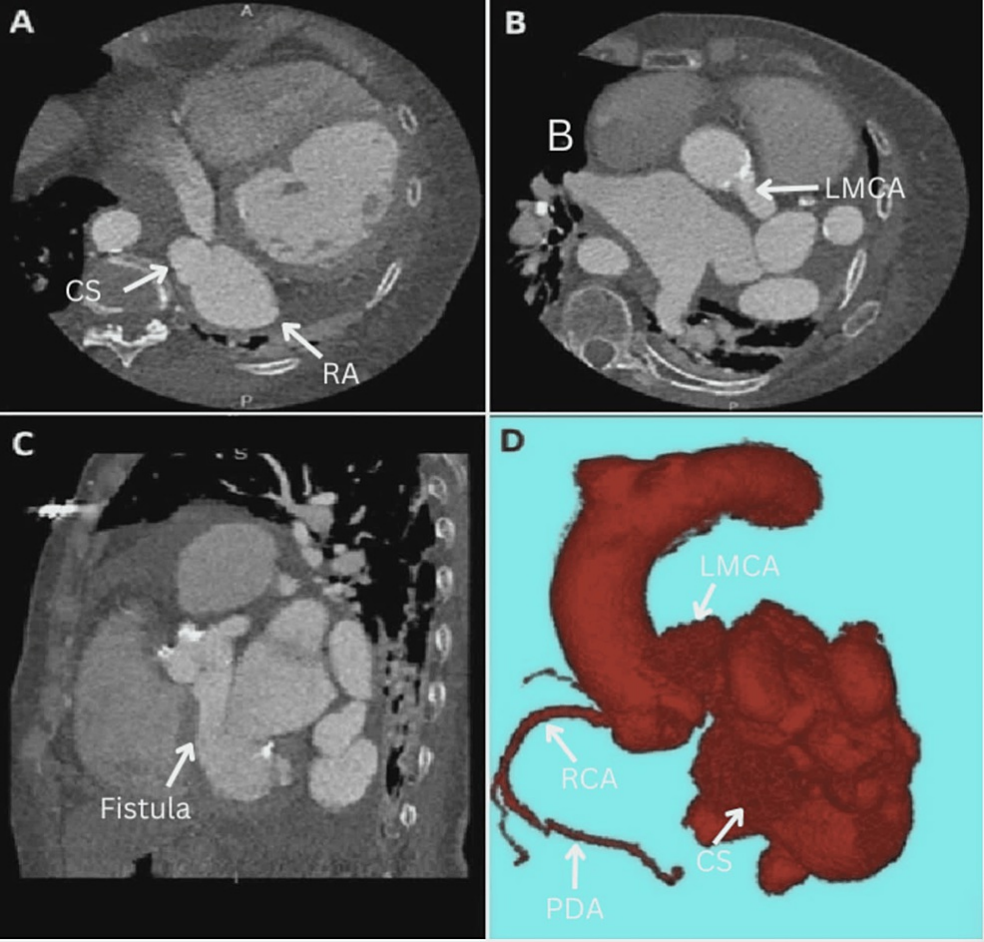
(A-C) Cardiac CTA. IV contrast is noted filling the right atrium during this arterial-timed contrast bolus study. The anatomy of the left coronary artery and circumflex is unable to be interpreted. (D) 3D CTA rendering. The coronaries arise from a normal position. There is right dominance. The left main coronary artery arises from the left coronary cusp and appears markedly dilated and tortuous. There is a large cavernous and partly calcified coronary arteriovenous fistula from the left main artery that appears to drain to the right atrium via a dilated coronary sinus. Multiple loops and segments of this large dilated fistula are noted along the lateral side of the left atrium and left ventricle as it courses toward the coronary sinus and right atrium. LMCA: Left main coronary artery; CTA: Computerized tomography angiography.

The right coronary arteries were normal, and the patient had a right descending aorta. The left coronary arteries could not be visualized due to the fistula, and cardiac computerized tomography angiography (CTA) was obtained for better visualization (Figure 4).

The cardiac CTA demonstrated a right dominant coronary system arising from their respective coronary cusps. The left main coronary artery was tortuous and dilated, which made the anatomical evaluation of the left descending artery and left circumflex artery challenging. The difficulty in interpreting this vasculature stemmed from the finding of the large arteriovenous calcified cavernous fistula arising from the left main coronary artery and draining into a dilated right coronary sinus. Multiple segments and loops of this dilated fistula were noted to be coursed along the lateral side of the left atrium and left ventricle before draining into the right atrium and coronary sinus, confirming the diagnosis. The WATCHMAN procedure was canceled due to the large size of the coronary arteriovenous fistula and foreseen complications.

## Discussion

Patients with CAVFs may be asymptomatic, but they can also present with symptoms such as dyspnea on exertion and unstable angina, as observed in our case [1,5]. Risk factors for developing CAVFs include trauma, diseases such as acute myocardial infarction, or iatrogenic causes such as percutaneous coronary intervention [1,6]. While the co-occurrence of gastrointestinal AVM and coronary AVM is not well-documented in the literature, this case involves a patient with a history of gastrointestinal AVM and colon cancer, raising questions about the etiology.

In this case, the patient has a history of gastrointestinal AVM and colon cancer status post partial colon resection. Blunt abdominal trauma can lead to arteriovenous fistula formations, but it is unclear if that could have developed in relation to her abdominal surgery or if there was a genetic component [7]. CAVFs are commonly congenitally acquired due to retained primitive embryonic sinusoids causing fistula formation if not obliterated [7].

In our case, the patient could have congenitally developed CAVFs as she was found to have a coronary artery fistula at a rural hospital on two CT abdominal exams in 2018 and 2021, and on a CT chest exam two months before presentation. Alternative proposals for the mechanism of post-natal AVM formation have been described, including angiopathic response to ischemia and inflammation, proliferative response to epigenetic modifications, and response to physical stress from hemodynamic forces [8,9]. Regardless of etiology, clearer communication of this finding between the primary care provider and the heart team could have minimized excessive cardiac interventions by foregoing workups for contraindicated procedures.

Treatment for CAVFs depends on several factors, including the size and location of the fistula, the severity of symptoms, and the patient’s overall health. Asymptomatic patients with small fistulas may be managed conservatively with regular monitoring and imaging to track the fistula’s characteristics over time. Symptomatic patients, or those with larger fistulas, typically require active treatment to prevent complications such as heart failure, arrhythmias, myocardial ischemia, and endocarditis.

Medical management may involve pharmacotherapy to control symptoms and mitigate the risk of heart failure. This includes using beta-blockers or calcium channel blockers to manage heart rate and myocardial oxygen demand as well as diuretics to address heart failure symptoms [2].

Transcatheter closure is often the preferred approach for patients requiring more definitive treatment. This minimally invasive procedure involves the use of devices such as coils, plugs, or covered stents to occlude the fistula. Transcatheter closure is favored due to its lower risk profile and quicker recovery time compared to surgical options. The success of this procedure depends on the fistula’s anatomy being suitable for percutaneous access and intervention.

Surgical intervention, including direct ligation or resection of the fistula, is reserved for cases where the fistula is large, complex, or inaccessible via catheter-based techniques. Surgery may also be indicated when there is a significant risk of complications from the fistula, such as myocardial ischemia or coronary artery aneurysms.

The prognosis for patients undergoing treatment for CAVFs is generally favorable, particularly when the condition is diagnosed early and managed appropriately. Transcatheter closures have shown high success rates with low complication rates, while surgical interventions, though more invasive, also yield positive outcomes. Challenges in treating CAVFs include the anatomical complexity of some fistulas, necessitating advanced imaging for precise procedural planning, and the need for careful patient selection to balance the risks and benefits of intervention [6].

In addition to these management options, considering the suggestion to utilize ECG-gated multidetector computed tomography for assessing CAVFs would be beneficial. This non-invasive approach can effectively demonstrate the origin, draining chamber, and size of CAVFs, potentially eliminating the need for invasive diagnostic procedures like coronary catheterization, especially in low-risk coronary artery disease (CAD) patients. This not only aids in diagnosis and preoperative planning but also helps rule out CAD, offering a valuable diagnostic alternative [10].

Considering our patient's advanced age, the presence of multiple comorbidities elevating the procedural intervention risk, and minimal symptomatology, the choice was made to forego surgical intervention. Presently, the patient's dyspnea ameliorated with medical interventions, including adherence to a low-sodium diet regimen and 40 mg oral administration of furosemide. She had to maintain regular follow-up appointments with the structural heart team, undergoing TEE every three to five years to meticulously track disease advancement.

## Conclusions

The occurrence of CAVFs, an uncommon cardiac irregularity, is particularly infrequent among older adults. Typically, it is discovered by chance when investigating symptoms such as shortness of breath or chest pain, where coronary angiography is necessary to determine the most effective treatment strategy. Most of the time, fistulas do not show any noticeable symptoms or only produce minor nonspecific symptoms and can therefore remain undetected throughout a person's life. The known association between malignancy and non-coronary AVM and the possibility of post-natal AVM development support consideration of the possibility of similar pathophysiology contributing to late CAVF presentations. This case highlights the possible utility of evaluating CAVFs in patients with a history of gastrointestinal AVMs who similarly present with clinical symptoms of high-output heart failure and coronary steal syndrome. Once identified, this could simplify the treatment approach and improve communication between healthcare providers to minimize the risk of harm to the patient.
